# Hepatitis E virus infection is highly prevalent among pregnant women in Accra, Ghana

**DOI:** 10.1186/1743-422X-6-108

**Published:** 2009-07-20

**Authors:** Andrew A Adjei, Yao Tettey, John T Aviyase, Clement Adu-Gyamfi, Samuel Obed, Julius AA Mingle, Patrick F Ayeh-Kumi, Theophilus K Adiku

**Affiliations:** 1 Department of Pathology, University of Ghana Medical School, Accra, Ghana; 2 Department of Microbiology, University of Ghana Medical School, Accra, Ghana; 3 Kumasi South Hospital, Kumasi, Ghana; 4 Department of Obstetrics and Gynaecology, University of Ghana Medical School, Kumasi, Ghana; 5 Komfo Anokye Teaching Hospital, Kumasi, Ghana

## Abstract

**Background:**

Hepatitis E virus (HEV) is highly endemic in several African countries with high mortality rate among pregnant women. The prevalence of antibodies to HEV in Ghana is not known. Therefore we evaluated the prevalence of anti-HEV IgG and anti-HEV IgM among pregnant women seen between the months of January and May, 2008 at the Obstetrics and Gynaecology Department, Korle-Bu Teaching Hospital, Accra, Ghana.

**Results:**

One hundred and fifty-seven women provided blood samples for unlinked anonymous testing for the presence of antibodies to HEV. The median age of participants was 28.89 ± 5.76 years (range 13–42 years). Of the 157 women tested, HEV seroprevelance was 28.66% (45/157). Among the seropositive women, 64.40% (29/45) tested positive for anti-HEV IgM while 35.60% (16/45) tested positive to HEV IgG antibodies. HEV seroprevalence was highest (46.15%) among women 21–25 years of age, followed by 42.82% in = 20 year group, then 36.84% in = 36 year group. Of the 157 women, 75.79% and 22.92% were in their third and second trimesters of pregnancy, respectively. Anti-HEV antibodies detected in women in their third trimester of pregnancy (30.25%) was significantly higher, P < 0.05, than in women in their second trimester of pregnancy (25.0%).

**Conclusion:**

Consistent with similar studies worldwide, the results of our studies revealed a high prevalence of HEV infection in pregnant women.

## Background

Hepatitis E virus (HEV) infection is a major cause of human viral disease with clinical and pathological features of acute hepatitis. The infection represents an important public health concern in many developing countries, where it is primarily transmitted through the faecal oral route due to contaminated water and food [1] and is often responsible for epidemic outbreaks [2]. The infection affects primarily young adults and is generally mild [3]; however, the mortality rate is higher among women, especially during the second or third trimesters of pregnancy [4-6]. In Sudan, a case:fatality ratio of 17.8% was found in an outbreak in Darfur, with a ratio of 31.1% among pregnant women [7]. In related studies, Stoszek *et al*. and Patra *et al*. reported prevalence rates of 84.3% and 60%, among pregnant women in Egypt and India, respectively [8,9].

In Ghana, studies of HEV seroprevalence in pregnant women have not been done previously. However, recently we observed HEV seroprevalence rate of 38.1% among persons who work with pigs in Ghana (Unpublished data). In a much earlier related study, Martinson *et al*. [10] reported the seroprevalence of HEV among children in rural Ghana to be 4.4%. Many HEV outbreaks have been reported in Africa [11-16], such as Uganda in 2007–2008 [11], and Sudan and Chad in 2004–2005 [15].

Ghana, an area of endemicity for viral hepatitis B and C, has never had an epidemic of hepatitis E. However, recent reports indicate cases of acute hepatitis without a defined aetiology (Unpublished data, Department of Medicine and Therapeutics, Korle-Bu Teaching Hospital [KBTH], Accra). Currently in Ghana, pregnant women are not screened routinely for HEV antibodies. The present study was conducted to determine the seroprevalence of HEV among pregnant women in Ghana and to assess the implications for antenatal screening.

## Subjects and methods

### Study area and subjects

This study was conducted at the Obstetrics and Gynaecology Outpatient Clinic of the KBTH, Accra, Ghana, between January and May 2008. KBTH, situated in the nation's capital, Accra is the leading tertiary hospital and the major referral center in the country. Hence, most of the patients seen at KBTH are referral cases from various parts of Southern Ghana. It also serves as the Teaching Hospital of the University of Ghana Medical School (UGMS), Accra, Ghana. Thus the demographics of the patients that were tested in this study were not limited to a specific social group. The patients in this study originated from various social and ethnic groups as well as geographically distinct areas from the vast territory of the Greater Accra region and the Southern part of Ghana. There was no selection of patients from a larger cohort of cases; the cases presented here were the first 157 consecutive patients to enter the Gynaecology Clinic during the period of the study who consented to participate in the study and a request for HEV testing was sought. The samples used for the study were the excess sera from blood samples drawn from these 157 pregnant women for their routine antenatal (syphilis, ABO and Rhesus, hepatitis B virus [HBV], hepatitis C virus [HCV] and human immunodeficiency virus [HIV]) testing, with all identifiers removed except for age, were assayed for antibodies to HEV. All pregnant women who simultaneously or unilaterally tested positive for HBsAg, HCV, HIV and syphilis were excluded from the study. The study was reviewed and approved by the Ethical and Protocol Review Committee of the UGMS.

### Sample Collection and Serological Tests

Venous blood samples were taken and sera were separated and kept frozen at -20°C before being sent to our laboratory for testing. Fully informed consent was obtained from each study subject. When study subjects were younger than 18 years, informed consent was obtained from their parents. Samples were anonymous for the patient's name and hospital number, but data on age were retained. All of the sera were screened in duplicate for antibodies (IgG and IgM) to HEV using ELISA Kit (International Immuno-Diagnostics, CA, U.S.A.) in accordance with the manufacturer's instructions. The results were scored as positive or negative according to the standard procedures recommended by the manufacturer. Positive and negative controls were included in all the ELISA microplates assayed.

### Statistical analysis

The Statistical Analysis System version 9.1 (SAS Institute) was used to complete all data analysis. We divided the pregnant women into five categories of age: ≤ 20, 21–25, 26–30, 31–35, and = 35 years. Serum samples were classified as positive or negative. In the univariate analysis, the frequency for each of the age categories and the mean, median and maximum and minimum age for the overall sample were determined, as well as SD. We repeated the univariate analysis of age after having stratified the data by serum analysis results and compared the mean ages for a statistically significant differences using Student's *t*-test. We also obtained the frequency of seropositive and seronegative women. In the bivariate analysis, we evaluated the relationship between the age and serum results categories using Pearson's χ^2 ^test. Logistic regression analysis was used to model the relationship between age categories and serum results. The logistic model with a maximum-likelihood estimate was fitted to the ordinal response of age categories and 95% confidence intervals for odd ratios were calculated with the age category of ≤ 20 years as the reference group. A χ^2 ^test for trend over increasing age categories was also performed.

## Results

A total of 157 pregnant women were screened for the presence of anti-HEV IgG and anti-HEV IgM antibodies. Their ages ranged from 13 to 42 years, with a mean age ± SD of 28.89 ± 5.76 years. The median and modal ages of all of the pregnant women studied were 29 years. All of the patients were found to be healthy on routine antenatal medical examination and all of the serum samples were assayed in duplicate.

Overall, the HEV sero-prevalence rate among pregnant women at the KBTH in Accra, Ghana over the 5 month period was 28.66% (45 out of 157). Of the seropositive pregnant women, 64.40% (29 out of 45) tested positive for anti-HEV IgM whereas 35.60% (16 out of 45) tested positive for anti-HEV IgG.

The age distribution of pregnant women seropositive for HEV ranged from 18 to 38 years and their median and modal ages were 35 and 37 years, respectively. The age distribution of pregnant women seronegative for HEV ranged from 15 to 41 years and both their median and modal ages were 26 years. The mean age (± SD) of the seropositive pregnant women (32.40 ± 6.00 years) was significantly higher (P < 0.0001) than that of the mean age of the seronegative pregnant women (25.60 ± 5.80 years).

As shown in Table 1, the overall prevalence rate of antibodies to HEV was highest (46.15%) among pregnant women 21–25 years of age, followed by 42.82% in ≤ 20 year group, then 36.84% in ≥ 36 year group. There was no correlation between increasing age and HEV sero-positivity.

**Table 1:** Odd ratios for HEV Seropositivity and corresponding 95% confidence intervals (CI) by age of pregnant women in Accra, Ghana.

**Age(years)**	**Women (n)**	**HEV-Positive Women [n (%)]**	**Odd ratios**	**95%CI**
≤20	14	(42.82)	2.75	0.85–8.95

21–25	26	12(46.15)	1.41	0.51–3.90

26–30	58	14(24.10)	1.26	0.55–2.86

31–35	40	6 (15.00)	0.18	0.04–0.77

≥36	19	7 (38.84)	1.21	0.39–3.94

Of the 157 women, 119 (75.79%) were in their third trimester of pregnancy (gestational period of 31 + 4.6 weeks) while 36 (22.92%) were in their second trimester (gestational period of 22 + 3.3 weeks). Only two patients were in their first trimester (6 weeks) of pregnancy. HEV seroprevalence detected in women in their third trimester of pregnancy (30.25%; 36 out of 119) was higher, than in women in their second trimester of pregnancy (25.0%, 9 out of 36). However, in bivariate analysis, anti-HEV reactivity was positively associated with the stage of pregnancy (OR 1.34; CI, 0.58–3.13). Women in their first trimester of pregnancy were negative for both IgG and IgM anti-HEV antibodies.

A similar pattern of positive association (OR 2.19; CI, 0.76–6.29) of anti-HEV reactivity with education was found among pregnant women (Table 2). Anti-HEV reactivity among women with no formal education (43.75%, 7 out of 16) was higher than that of their counterparts with primary or basic (28.04%; 23 out of 82), secondary (27.50%, 11 out of 40), and tertiary (15.78%; 3 out of 19) level of education. There was no statistical difference between them, P > 0.05.

**Table 2:** Odd ratios for HEV Seropositivity and corresponding 95% confidence intervals (CI) by stage of pregnancy and level of formal education attained by pregnant women in Accra, Ghana.

**Stage of Pregnancy**	**Women (n)**	**HEV-Positive women [n(%]**	**Odd ratios**	**95%CI**
First trimester	2	0 (0)	0.00	-

Second trimester	36	9 (25.00)	0.82	0.35–1.92

Third trimester	119	36(30.25)	1.34	0.58–3.13

**Formal Education**				

Basic	82	23(28.05)	1.00	0.50–2.01

Senior High	40	11(27.50)	0.97	0.43–2.15

Tertiary	19	3 (15.79)	0.44	0.12–1.61

None	16	7 (43.75)	2.19	0.76–6.29

The prevalence rate of antibodies to HEV was highest (40%; 2 out of 5) among pregnant women who were students, followed by 35.29% (18 out of 51) in women engaged in petty trading at the market places, then 26.66% (4 out of 15) in the unemployed group, and 23.80% (20 out of 84) in women engaged in fashion and design. Two of the pregnant women were engaged as housewives and none of them tested positive for antibodies to HEV (IgG and IgM).

## Discussion

To our knowledge, this is believed to be the first study to determine the prevalence of HEV infection in pregnant women in Ghana, and demonstrates the high prevalence of and the considerable potential for the transmission of HEV infection in pregnant women in Ghana. Although there is no report from the Ministry of Health, Accra, Ghana indicating that Ghana is an endemic area for hepatitis E, this study found very high overall prevalence rates (28.66%) of HEV antibody among pregnant women, suggesting the possibility of subclinical infections in the country. The finding of higher HEV antibody prevalence among pregnant women attending the Obstetric Outpatient Clinic of the KBTH, Accra, Ghana is consistent with literature, and is widely attributable to poor sanitation and contamination of the water supply [1,17]. The overall seroprevalence of HEV infection among the pregnant women in Ghana (28.66%) is higher than the results of similar studies done in the United Arab Emirates (20%; [18]), Gabon (14.1%; [19]) but lower than the sero-prevalence of HEV infection among pregnant women in Egypt (84.3%; [8]), Ethiopia (59%; [20]) and Sudan (31.1%; [7]). The high seroprevalence of HEV in pregnant women at the KBTH may suggest that HEV may be widespread among pregnant women in the country and therefore reasonable to speculate that HEV may circulate in the general population and this calls for population-based study to confirm this speculation. In addition, because the virus is transmitted through the faecal-oral route, transmission of HEV is greatly dependent on the sanitary conditions under which the pregnant women live and work. In Ghana, there are great social differences and sanitary conditions are quite precarious in many areas. Majority (146/157; 93.0%) of the pregnant women live and work in densely populated areas where the sanitary conditions are very deplorable and also where animals, such as, sheep, goats, cows, dogs, rats, and cats share their habitat with humans. In fact, serum anti-HEV antibodies have been found in domestic animals such as rats, sheep, dogs, cats and may serve as reservoirs for the transmission of human hepatitis E [21-23].

Growing evidence suggest that the seroprevalence of antibodies to HEV is higher among women in their third trimester of pregnancy [4,24-26]. Similar results were obtained in our study. There was a significant preponderance of HEV infection in the third trimester of pregnancy (35 out of 119; 29.41%) compared to women in their second (9 out of 36; 25%) and first (0 out of 2; 0%) trimesters of pregnancy.

Of the pregnant women, only two were engaged as housewives and interestingly both of them tested negative for antibodies to IgG anti-HEV and IgM anti-HEV in comparison to those engaged in buying and selling at the market (18 out of 51; 35.29%), fashion and design (20 out of 84; 23.8%), and unemployed (4 out of 15; 26.66%). The reason(s) for this disparity cannot be discerned from our study and further studies need to be done to define the low and high prevalence rates of anti-HEV antibodies in such populations. There is also the need for further studies to define the clinical and epidemiological importance and pathogenesis of HEV infection among pregnant women engaged in different occupations.

The policy of not screening for HEV antibodies in pregnant women and in blood and organ donors in most countries is based partly on its perceived low prevalence and on the **low **life time risk of its associated diseases, although the cost of antenatal and blood-donor screening could be limited by selecting those thought to be at high risk. With appropriate counseling, screening for HEV should be accepted in the same light as testing for HIV, which recently has been recommended as part of the routine antenatal screening programme for several countries [26]. However, unlike HIV infection, infection with HEV is less likely to become clinically apparent and the factors that confer a high risk of developing associated disease have not been fully defined. In the meantime, antenatal screening of pregnant women would ensure that doctors could take further precautions to protect against nosocomial infection and to ensure that newborns do not swallow blood at the time of delivery from HEV-seropositive mothers, in order to minimize perinatal HEV transmission. The argument for antenatal HEV testing in Ghana is compelling, because of the precarious sanitary conditions in most urban and rural areas, increased incidence of acute viral hepatitis without a defined aetiology (unpublished data, Department of Medicine and Therapeutics, KBTH), and the high infant and maternal mortality. Our findings re-emphasize the suggestion that targeting high-risk women or universal testing in high prevalence areas, which includes Ghana, could identify most women infected with HEV at a relatively low cost [1,4].

In conclusion, the results of this study and our recent results in persons who work with pigs and blood donors (unpublished data) demonstrate a high prevalence of HEV infection in Ghana. Therefore, preventive measures to decrease the spread and transmission of HEV are warranted. These measures should include the systematic HEV screening of pregnant women in order to counsel them about the risk of contracting and transmission of HEV. However, the findings and conclusions of this study are limited by the small sample size of pregnant women. A further larger-scale prospective survey of HEV infection among pregnant women in Ghana should be conducted to validate our findings and to analyse in more detail the clinical and the epidemiological features of this infection and to evaluate the cost-effectiveness of antenatal HEV screening in Ghana.

## Consent

Fully informed consent was obtained from each study subject. When study subjects were younger than 18 years, informed consent was obtained from their parents.

## Competing interests

The authors declare that they have no competing interests.

## Authors' contributions

AAA, YA, JTA, CAG, SO, JAAM, PFAK, TKA conceived of the study, participated in its design and coordination. All authors read and approved the final manuscript.
